# Spatially resolved investigation of all optical magnetization switching in TbFe alloys

**DOI:** 10.1038/s41598-017-09615-1

**Published:** 2017-08-25

**Authors:** Ashima Arora, Mohamad-Assaad Mawass, Oliver Sandig, Chen Luo, Ahmet A. Ünal, Florin Radu, Sergio Valencia, Florian Kronast

**Affiliations:** 1 Helmholtz-Zentrum Berlin für Materialien und Energie, Albert-Einstein Str. 15, 12489 Berlin, Germany; 2 Institut für Experimentalphysik, Freie Universität Berlin, Arnimallee 14, 14195 Berlin, Germany; 3 Department of Physics, University of Regensburg, Universitätsstraße 31, 93053 Regensburg, Germany; 4 Present Address: Max Born Institute for Nonlinear Optics and Short Pulse Spectroscopy, Max-Born-Straße 2A, Berlin, 12489 Germany; 5 Institut für Physik und Astronomie, Universität Potsdam, Karl-Liebknecht-Str. 24/25, 14476 Potsdam, Germany

## Abstract

Optical control of magnetization using femtosecond laser without applying any external magnetic field offers the advantage of switching magnetic states at ultrashort time scales. Recently, all-optical helicity-dependent switching (AO-HDS) has drawn a significant attention for potential information and data storage device applications. In this work, we employ element and magnetization sensitive photoemission electron microscopy (PEEM) to investigate the role of heating in AO-HDS for thin films of the rare-earth transition-metal alloy TbFe. Spatially resolved measurements in a 3–5 μm sized stationary laser spot demonstrate that AO-HDS is a local phenomenon in the vicinity of thermal demagnetization in a ‘ring’ shaped region. The efficiency of AO-HDS further depends on a local temperature profile around the demagnetized region and thermally activated domain wall motion. We also demonstrate that the thickness of the film determines the preferential switching direction for a particular helicity.

## Introduction

The direct control of magnetization using light at ultrashort timescales has the potential to revolutionize the future magnetic data storage technologies. Ultrafast switching times and reliable switching of magnetic states in high storage density devices are crucial for this purpose^1^. The very first attempt to use light as a stimulus for altering the magnetization was carried out to study the ultrafast demagnetization dynamics in nickel^2^. Ever since then, many experiments on magnetic materials have explored ultrafast demagnetization and all optical switching. Stanciu *et al*. were the first to demonstrate AO-HDS in which a circularly polarized femtosecond laser pulse switches the magnetization of a ferrimagnetic GdFeCo thin film as function of laser helicity^3^. Somewhat later it was revealed that the magnetization in GdFeCo can even be switched merely by a heat stimulus^4^. While GdFeCo seems to be a special case in which all-optical switching can occur from different demagnetization time scales of its magnetic sublattices^5, 6^, more recent studies revealed that AO-HDS is a general phenomenon existing in magnetic materials ranging from RE-TM ferrimagnetic alloys, multilayers, hetero-structures to even ferromagnetic films^7, 8^. This intriguing possibility of ultra-fast magnetization switching in high anisotropic ferromagnetic alloys such as CoPt and FePt has attracted significant interest for practical implications. There have been numerous debates about the microscopic origin of AO-HDS in ferromagnets or ferrimagnetic alloys^3, 7–9^. Most renowned in literature is the concept of momentum transfer via Inverse Faraday Effect (IFE)^6, 10, 11^ and the concept of preferential thermal demagnetization for one magnetization direction by heating close to T_c_ (Curie temperature) in the presence of magnetic circular dichroism (MCD)^9, 12–14^. Irrespective of proposed microscopic mechanisms, AO-HDS is observed to be a stochastic and cumulative process occurring in a narrow window of laser fluence above the threshold fluence^14–16^. The minimum number of pulses required to observe the switching further depend on the laser fluence, repetition rate, ambient temperature and the properties of the magnetic media in consideration.

In this study, we investigate all-optical magnetic switching using a stationary femtosecond laser spot (3–5 µm) in TbFe alloys via photoemission electron microscopy (PEEM) and x-ray magnetic circular dichroism (XMCD) with a spatial resolution of approximately 30 nm. We spatially characterize the effect of laser heating and local temperature profile created across the laser spot on AO-HDS in TbFe thin films. We find that AO-HDS occurs only in a ‘ring’ shaped region surrounding the thermally demagnetized region formed by the laser spot and the formation of switched domains relies further on thermally induced domain wall motion. Our temperature dependent measurements highlight the importance of attaining T_c_, local temperature and temperature gradients for helicity-dependent switching. In addition, by investigating a series of samples with different Tb concentrations and film thicknesses, we demonstrate that the switching direction for a given laser helicity, inverts at a threshold film thickness. This could indicate the presence of two mechanisms contributing to AO-HDS in our samples. We discuss the possible contributions and important parameters which are required to attain a reliable and efficient helicity dependent switching process.

## Experimental Details

The RE and TM magnetic sublattices in Tb_x_Fe_1−x_ alloys are anti-ferromagnetically coupled to each other and exhibit a perpendicular magnetic anisotropy. At compensation temperature (T_CM_), the magnetization of the individual Fe and Tb sublattices cancel each other resulting in a zero net remanent magnetization. The total magnetization above and below T_CM_ is dominated by the magnetization direction of Fe and Tb sublattices, respectively. It is possible to select T_CM_ based on the composition and thickness dependence of T_CM_ in such ferrimagnetic alloys^17–19^. We fabricated a series of amorphous Tb_x_Fe_1-x_ films (See Supplementary Table S1) by the means of magnetron sputtering on transparent MgO substrates (0.5 mm thick) at room temperature. The samples have a thickness ranging from 10 nm to 80 nm and Tb concentrations of x = 0.22 and x = 0.30 with Ta (5 nm) buffer layer. The TbFe films are capped by 1.8 nm of Pt in order to prevent oxidation. In the following report, the samples will be referred by TbXY where X specifies the Tb concentration in % (either 22 or 30) and Y specifies the thickness of TbFe magnetic alloy in nm.

**Figure 1:**
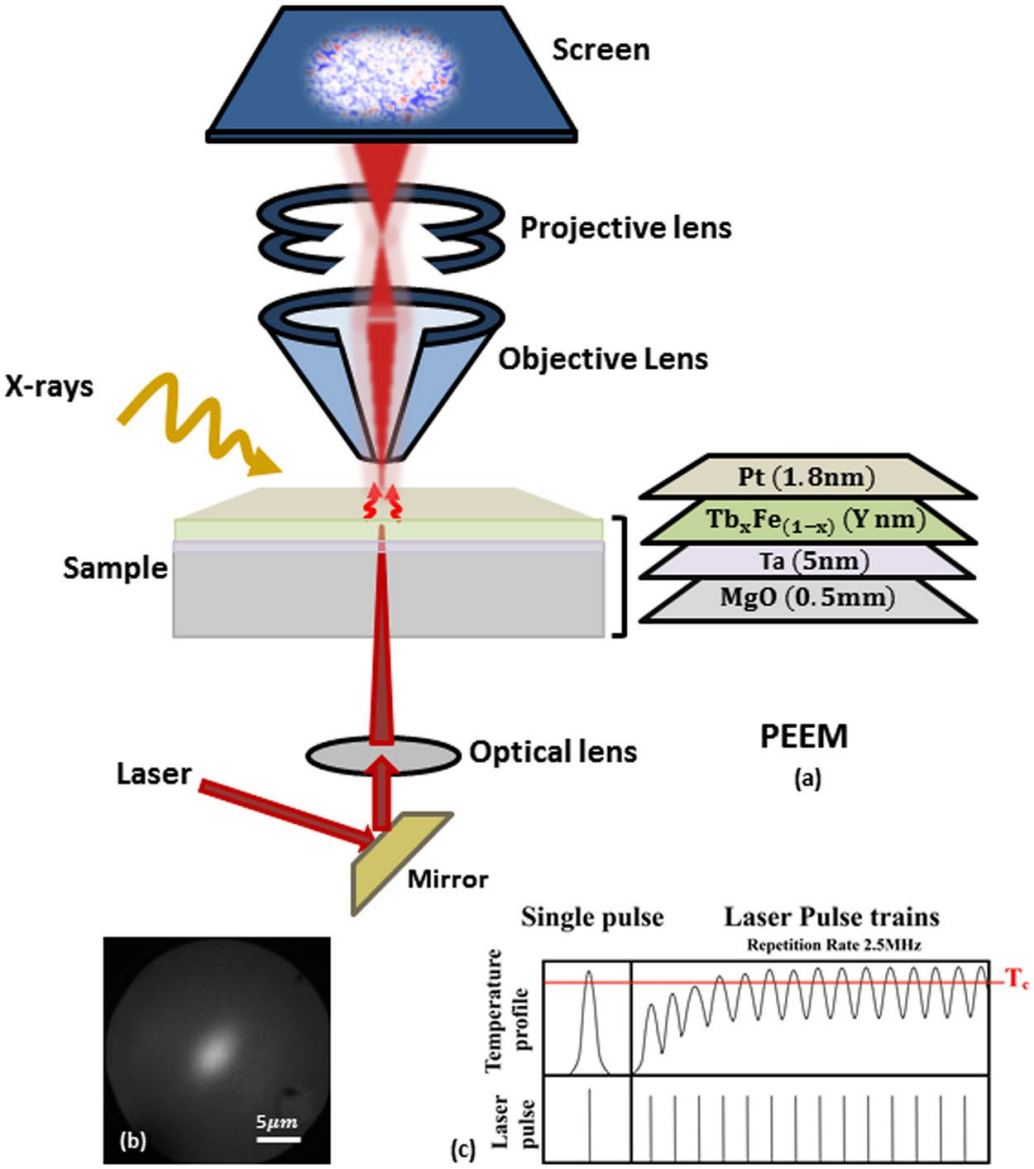
(**a**) Schematic of experimental setup for PEEM, the laser optics integrated sample holder and the sample. (**b**) Direct photoemission from the laser spot measured by PEEM showing Gaussian profile of the spot. (**c**) Schematic showing the laser profile and the corresponding temperature profile on the sample for a single laser pulse and multiple pulse trains.

Measurements are performed at UE49PGMa beamline at BESSY II Synchrotron facility in Helmholtz-Zentrum Berlin using the SPEEM station^20^ (based on an Elmitec III instrument). The PEEM provides a lateral resolution of 30 nm using x-rays which are incident at 16° to the sample surface. The absorption of x-ray emits photo-electrons from the sample which are under the influence of an accelerating potential of 10 kV between the sample and the objective lens. After passing through a series of projective lenses and a hemi-spherical energy analyzer with an energy resolution less than 0.2 eV, the lateral distribution of secondary electrons is imaged on a screen. Fig. 1(a) shows a schematic diagram of the experimental set up. Magnetic images are recorded at Fe *L*_3_ (706.6 eV) absorption edge, exploiting the XMCD for left and right circularly polarized x-rays (*σ*− and *σ*+). The XMCD magnetic contrast is calculated as the difference between the images taken by left and right circularly polarized x-rays normalized by their sum. The displayed XMCD contrast is proportional to the projection of the out-of-plane component of Fe magnetic moment along the beam propagation direction. The red/blue contrast in the XMCD images represents the two anti-parallel out-of-plane magnetic orientations and the white contrast either represents a domain wall, a non-magnetic state or an orthogonal magnetic orientation to the x-rays. We use a Ti-sapphire fs laser at a wavelength of 800 nm at which the MgO substrate is transparent to the laser. The obtained laser spot size measured with the PEEM is 3–5 µm (FWHM) (Fig. 1(b)). The laser can be operated in single-pulse or pulse-train mode at a repetition rate up to 2.5 MHz. Fig. 1(c) shows a schematic displaying the two modes and their expected temperature profiles build on the sample surface during laser exposure.

## Results and Discussion

**Figure 2:**
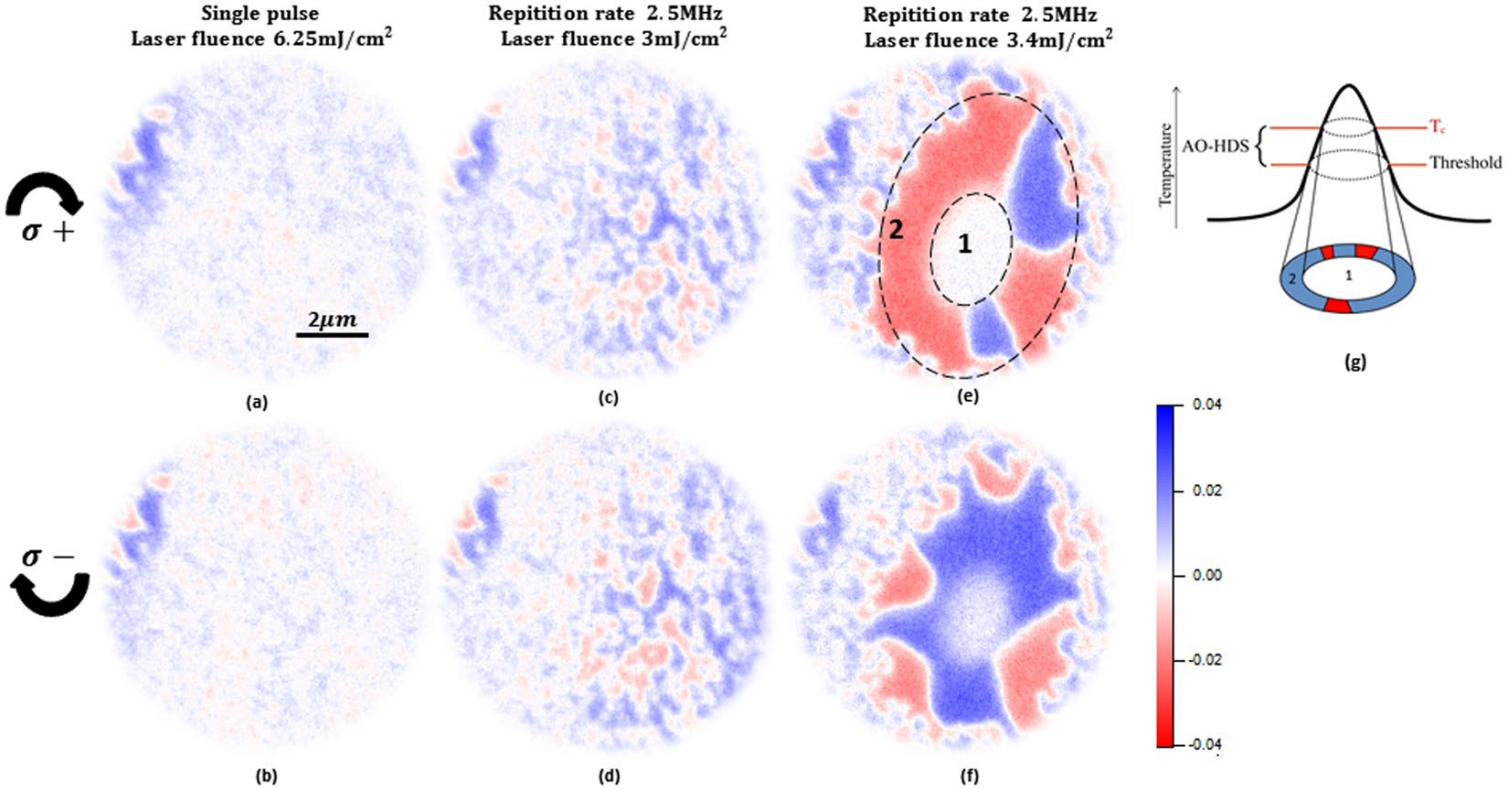
XMCD images showing magnetic contrast for (**a**,**b**) single shot measurements at opposite helicities.(**c**,**d**) Laser pulse trains applied below the threshold power at opposite helicities (**e**,**f**) laser pulse trains above threshold at opposite helicities showing AO-HDS. (**g**) Schematic showing laser profile and AO-HDS.

Previous experiments in similar TbFe films demonstrated that the laser induced switching is partially governed by dipolar fields^21–23^. To minimize this effect, we always initially demagnetize the laser spot area by a strong single laser pulse (6.25 mJ/cm^2^). Such high laser power leads to the formation of small domains as a result of thermal demagnetization as showed in XMCD images in Fig. 2(a) and (b). This procedure ensures zero stray fields from the surroundings which could have otherwise influenced the switching process. Fig. 2(a,b) show that we did not observe any significant helicity dependent effect induced by single laser pulses.

To study AO-HDS while the laser interacts with the sample, we applied laser pulse trains (as shown in Fig. 1(c)) with a repetition rate of 2.5 MHz. In this configuration, the temporal spacing between two consecutive pulses is 400 ns which is shorter than the thermal relaxation time of the sample. Therefore, a local heat profile builds up in the laser exposed region until an equilibrium between heat diffusion and heat accumulation by the laser is established^24, 25^. We record XMCD images in Fig. 2(c) and (d) while the sample is exposed to circularly polarized laser pulse trains of opposite helicities *σ*+ and *σ*−, respectively. The magnetic contrast in these XMCD images represents the temporal average over millions of consecutive laser pulses. Starting from a thermally demagnetized initial state and slowly increasing the laser fluence up to 3 mJ/cm^2^, we first observe an increase in magnetic domain size in Fig. 2(c) and (d) as compared to the demagnetized state in Fig. 2(a) and (b). This effect is independent of the laser helicity and can be attributed to thermally induced domain wall motion. By further increasing the laser fluence from 3 to 3.4 m J/cm^2^ we surpass the threshold of thermal demagnetization at the center of the laser spot where a region with zero magnetic contrast emerges (Fig. 2(e) and (f)). In that region the sample is getting at least temporarily demagnetized and possesses a random magnetization in between consecutive laser pulses which average to zero for the full laser pulse train (marked as 1). A schematic in Fig. 2(g) shows the projection of temperature profile of the laser on the affected sample region. While the magnetic contrast in the center region is quenched by thermal demagnetization we observe a ring-shaped region (marked as 2) appearing around it in which the preferential orientation of magnetization direction, displayed as red or blue contrast depends on the laser helicity, which we attribute to AO-HDS. An important observation from laser fluence-dependent measurements is that the onset of AO-HDS within the laser spot is concomitant with the occurrence of local thermal demagnetization, i.e. both effects seem to be directly linked. In a Gaussian laser spot, we observe AO-HDS as a local, fluence dependent phenomenon, occurring just at the threshold of thermal demagnetization occurring at T_c_.

The necessity to reach the proximity of T_c_ is qualitatively discussed by Gorchon *et al*. where they model AO-HDS as a combination of stochastic switching mechanism near T_c_ and MCD^14^. In addition, it has been shown that AO-HDS is altogether a cumulative heat dominated switching process requiring a certain number of pulses to occur^15^. In this context, the threshold fluence or the minimum number of laser pulses required for the switching process could be directly proportional to their ability to build a local temperature profile around T_c_. Our observation of limited extent of AO-HDS to a ring shaped region around thermally demagnetized area further supports this hypothesis. This behavior might be consistent for all class of materials such as ferromagnetic multilayers and hetero-structures including ferromagnets. Due to technical restraints of our experimental set up, we cannot sweep the laser spot across the sample, but we strongly believe that sweeping the laser spot would result in helicity dependent homogenously switched tracks written on the sample. At the end of these tracks, we would expect this thermally demagnetized region surrounded by the ‘ring’ as also observed in previous results^3, 7, 8^.

**Figure 3:**
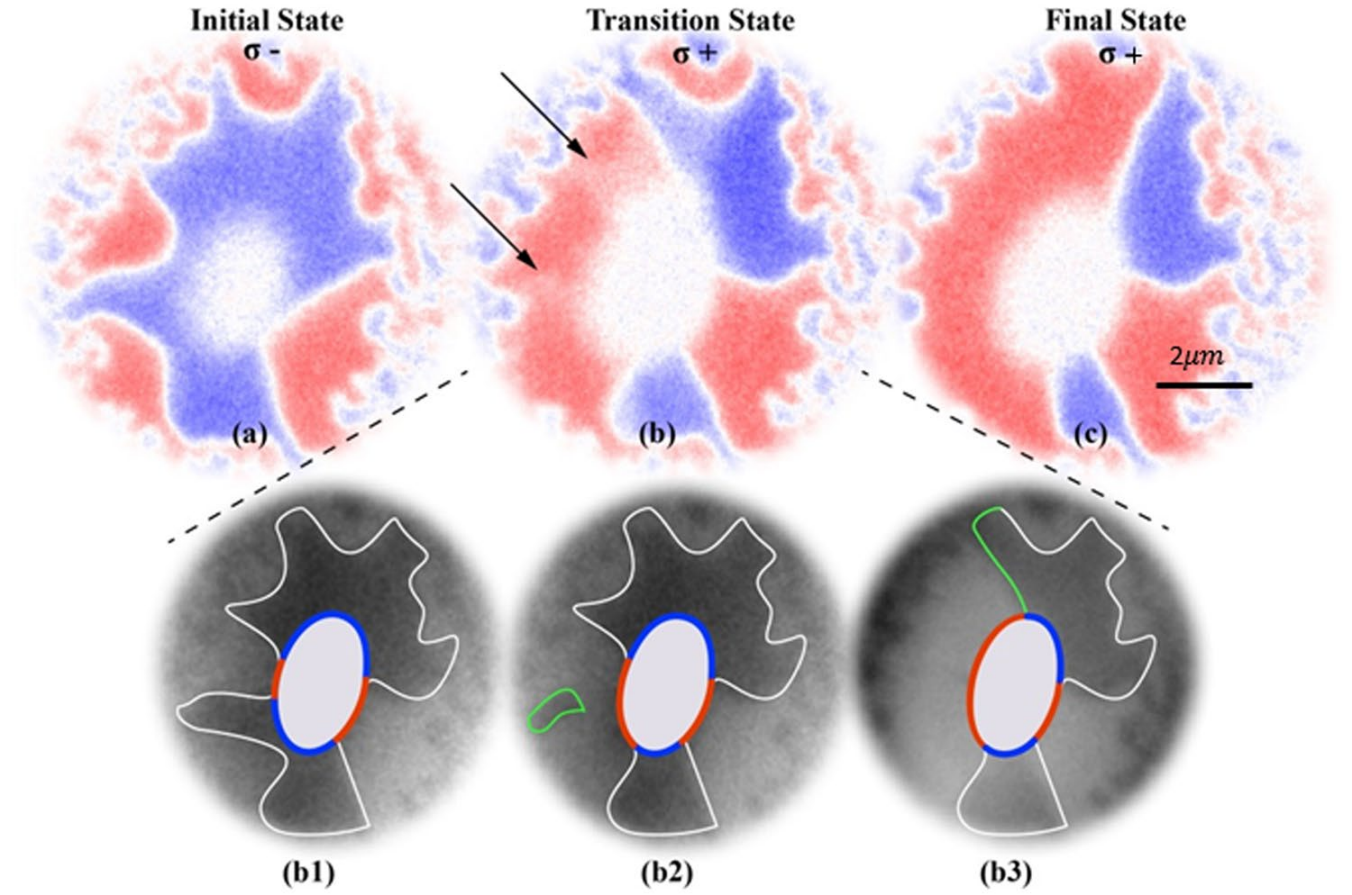
XMCD images showing magnetic domains around demagnetized region when laser helicity is flipped from **σ**− in (**a**) to **σ**+ in (**b**,**c**). Figure 3(b1–b3) show sequential thermally induced domain wall motion. White and green boundaries are to guide to the magnetic domains and recent changes with respect to previous image, respectively.

Our results provide direct evidence that AO-HDS occurs in the thermally activated region via domain wall motion. The local heat profile lowers the magnetic moment and increases the magnetic susceptibility. In such a state, a small stimulus is enough to promote the domain wall motion which can lead to formation of bigger and stable domains. Fig. 3 shows the evolution of magnetic domains during reversal of the laser helicity. Starting with mainly blue domain surrounding the demagnetized inner part of the laser spot in Fig. 3(a) we observe the growth of a mainly red domain after reversing the laser helicity in Fig. 3(b,c). The domain wall motion occurs in stochastic manner and sometimes the local magnetization reversal can take up to several seconds. Usually one XMCD image represents the average over several frames recorded with opposite helicity. In Fig. 3(b1–b3) we show the evolution of magnetic domains that has been extracted from single frames recorded with 3 seconds each to complete the XMCD image of Fig. 3(b). We can directly observe the growth of red domains at the expense of blue domains in the thermally activated area.

Although heat dominated domain wall motion has been considered as a limitation for deterministic switching in GdFeCo films^26^, the switching mechanism for our measurements in TbFe films instead requires heat induced domain wall motion in order to form large switched domains. At room temperature, the stimulus by the laser is not enough to switch the magnetization. The importance of thermal energy (hence the base temperature) has also been highlighted in a recent study^27^ where all-optical magnetic switching mechanism has been considered as a two-step process of laser-induced depinning and thermally activated domain wall motion. The switching in ferrimagnets like TbCo has been demonstrated as a ‘two step’ process where the first step leads to helicity independent multi-domain formation in 1ms followed by helicity dependent gradual re-magnetization within 100 ms^16^. This is in agreement with our findings that AO-HDS only occurs in the vicinity of T_c_ and thermally induced domain wall motion is an essential ingredient to switch larger domains by a stationary laser spot. Therefore the thermal gradient across the ring influences the width of the switched ring structures. In our setup the difference between the peak temperature reached by the laser and the base temperature determine the thermal gradient and hence the spatial extent of AO-HDS (See Supplementary Fig. S2(a,b)). In this context, heat conductivity across the sample could also be influential.

**Figure 4:**
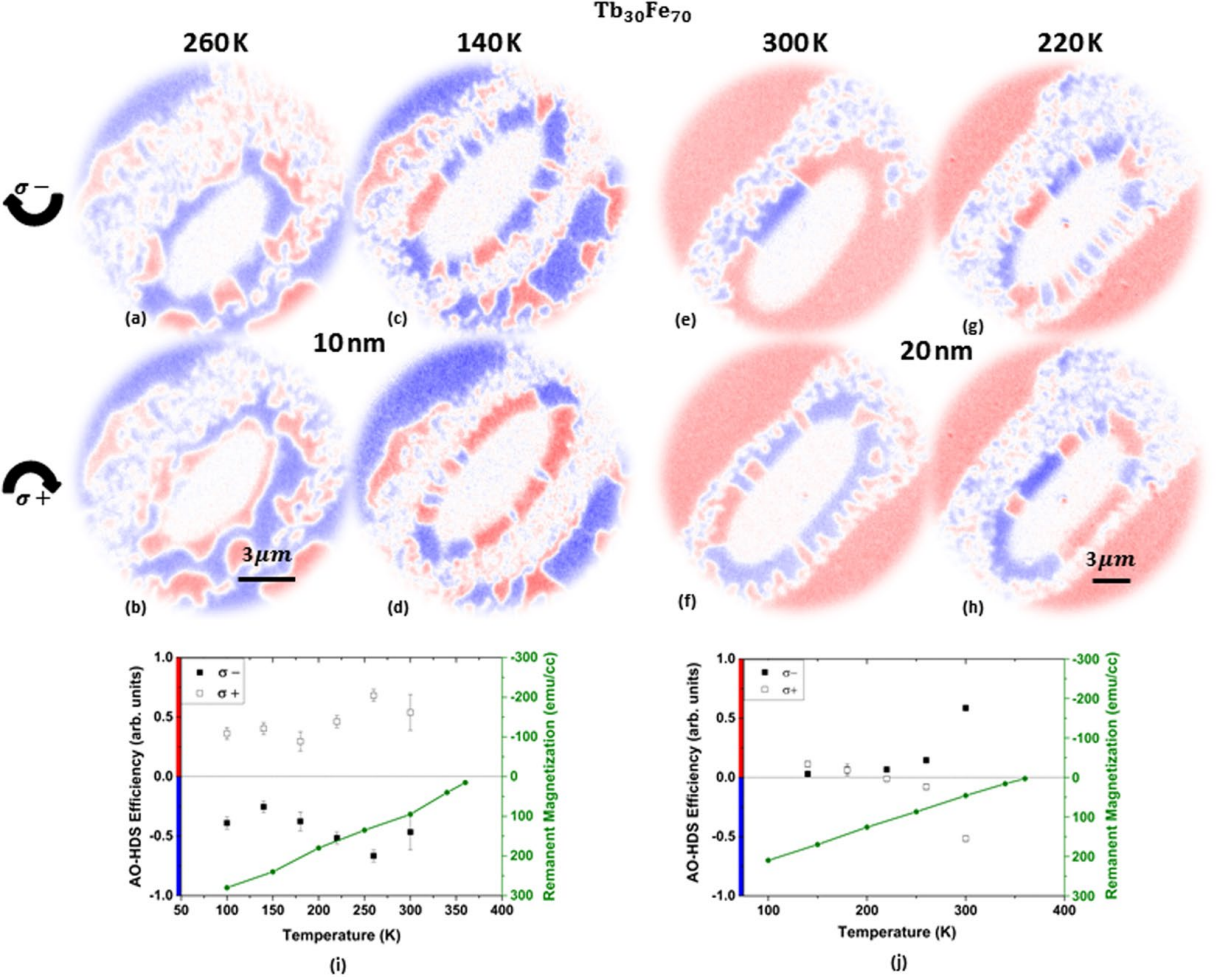
(**a**,**b)**, (**c**,**d**) XMCD images showing AO-HDS at 260 K and 140 K respectively for Tb3010 film. (**e**,**f**), (**g**,**h**) XMCD images showing AO-HDS at 300 K and 220 K respectively for Tb3020 film. (**i**,**j**) Plots showing the AO-HDS efficiency as a function of base temperature and the corresponding remanent magnetization (green axis) for Tb3010 and Tb3020, respectively.

To gain more insights about the influence of temperature, we investigated the impact of the sample base temperature on AO-HDS efficiency. Since the threshold fluence for AO-HDS decreases with increasing base temperature, we adapted the laser fluence in order to keep the maximum temperature reached at the center of laser spot constant. We ensured this by maintaining a constant spatial size of the thermally demagnetized region during each measurement. As mentioned before, prior to each measurement, we demagnetized the laser spot area by a single pulse of higher intensity to begin with a random multi-domain sate and minimize the contribution of dipolar field effects. Fig. 4(a–d) and (e–h) show the XMCD images recorded during laser exposure on Tb3010 and Tb3020, respectively. The switched ‘ring’ region is apparent in XMCD images in Fig. 4. Here, we would first like to address the switching dependence on temperature and later comment on the thickness dependent switching in the two samples.

We studied the switching efficiency for both the films in a temperature range from 100 to 300 K. Fig. 4 (a,b) and (c,d) show AO-HDS by laser of opposite helicities in a ring shaped region at 260 K and 140 K respectively for Tb3010. We can observe that efficiency of AO-HDS decreases at lower temperature where the uniform contrast in the switched ring region starts disintegrating into random magnetic domains. A similar behavior is observed for Tb3020 where Fig. 4(e,f) and (g,h) show AO-HDS by laser of opposite helicities in a ring shaped region at 300 K and 220 K respectively. To quantify the efficiency of AO-HDS at different temperatures, we evaluate the ratio of difference between the circumference of the ring covered in blue and red domains normalized to the total ring circumference:

$$Efficiency=\frac{{L}_{red}-{L}_{blue}}{{L}_{red}+{L}_{blue}}$$

where *L*_*red*_ and *L*_*blue*_ are the circumference of the ring covered by red and blue contrast respectively. An efficiency ratio of 1(−1) would imply that the whole ring is surrounded by red (blue) magnetization depicting a 100% AO-HDS. If the ratio is 0, this would imply a 50–50% distribution of red and blue magnetization accounting for a purely random switching behavior.

**Figure 5:**
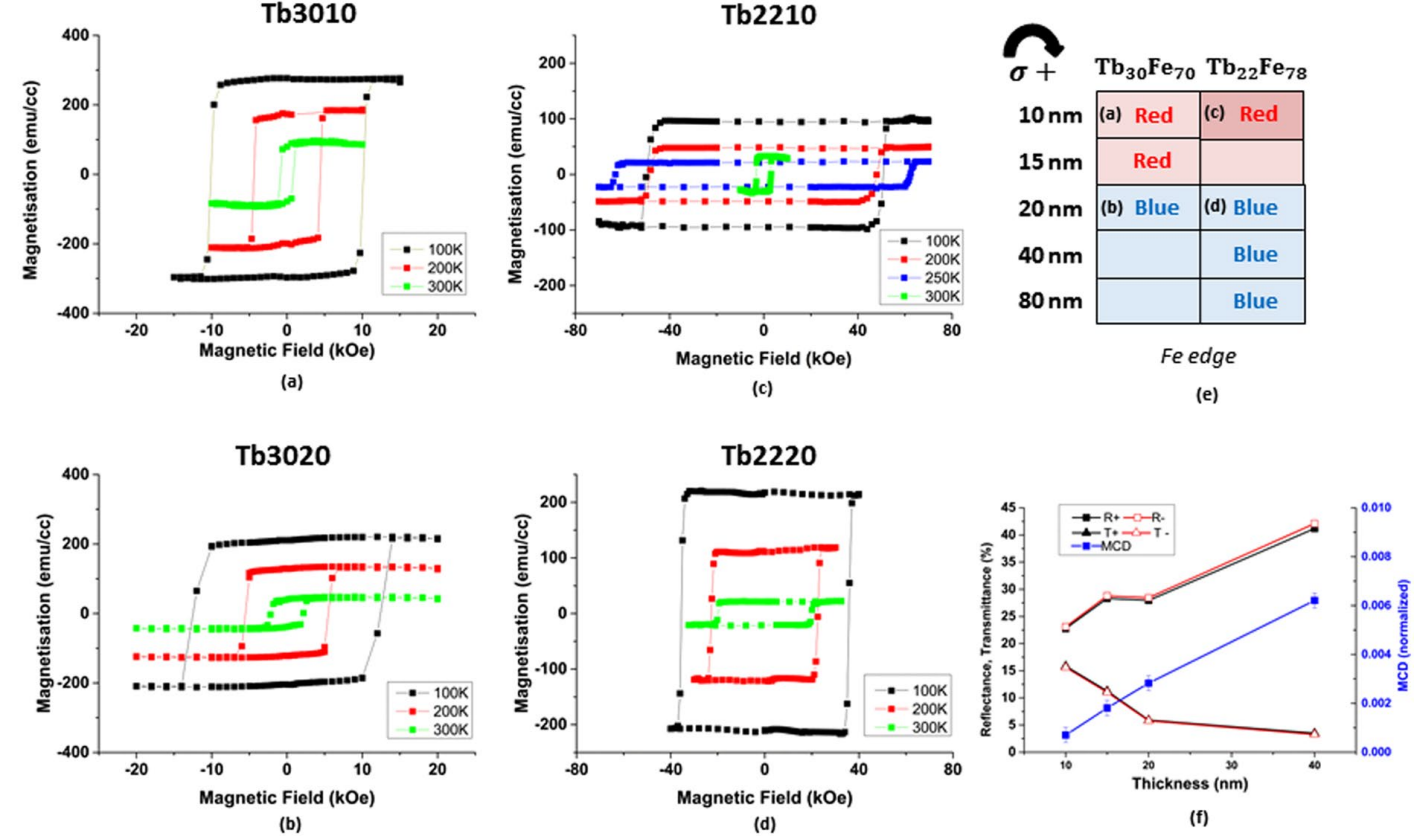
Hysteresis loops in out of plane configuration measured for (**a**) Tb3010 (**b**) Tb3020 (**c**) Tb2210 and (**d**) Tb2220 samples. (**e**) Figure showing the switching direction for different samples measured at Fe *L*_3_ edge for a fixed laser helicity. (**f**) Plot showing the measured reflectivity, transmission and asymmetry in absorbed laser power for opposite magnetization due to MCD effect as function of sample thickness.

We plotted the temperature dependent efficiency of AO-HDS in Fig. 4(i) and (j) for Tb3010 and Tb3020, respectively. Filled and the open squares represent the opposite laser helicities. It has been recently shown that a low remanent magnetic state of a sample is crucial for AO-HDS^28, 29^. In this context, we compare our efficiency data with remanent magnetization vs. temperature data measured by SQUID-VSM (Superconducting quantum interference device-vibrating sample magnetometer). The green axis in Fig. 4(i) and (j) shows the remanent magnetization data vs. temperature for Tb3010 and Tb3020, respectively. The remanent magnetization plotted here is extracted from the hysteresis loops measured by SQUID at different temperatures in out of plane configuration as depicted in Fig. 5(a) and (b). For Tb3020 film we find a reasonable agreement as its remanent magnetization decreases at higher temperature. However, for Tb3010, we do not observe a clear correlation between the temperature dependence of AO-HDS efficiency and remanent magnetization. This disagreement is not too surprising if we consider that AO-HDS occurs only in a corona-like region around the demagnetized region. Inside the ring region the temperature is at least temporarily after each laser pulse just below T_c_, thus the remanent magnetization at base temperature would have little impact on the switching behavior. Reducing the base temperature of the sample and simultaneously increasing the laser fluence enhances the temperature gradient around the laser spot. The temperature gradient may rather impact on domain size and domain wall mobility as well as on thermally induced domain wall motion. These parameters seem important to determine whether or not a stimulus by the laser close to T_c_, in terms of a small MCD or IFE effect, can establish or switch a magnetic domain. The impact of temperature on domain size and domain-wall mobility is shown in Supplementary Fig. S2(c,d).

The second important observation that can be made from Fig. 4 is that the magnetization reversal direction with a particular helicity is opposite for the two thicknesses. The switching direction in the ring region for a given laser helicity reverses from blue/red (Fig. 4(a,b)) to red/blue (Fig. 4(e,f)) as the thickness is increased from 10 to 20 nm. This is surprising as both samples have a Tb dominant sub-lattice with no compensation temperature and similar magnetic properties as evidenced by the SQUID data in Fig. 5(a,b). Please note that all our measurements start from a random multi-domain state. Thus even though the uniform background magnetization for the two samples is opposite (blue in Tb3010 and red in Tb3020), the switching direction is independent of the initial magnetic contrast. For a better understanding of this intriguing thickness dependence, we investigated the switching behavior of the samples Tb2210, Tb2220, Tb2240 and Tb2280 which have different Tb concentration and different magnetic properties. Out of plane hysteresis curves for Tb2210 and Tb2220 are plotted in Fig. 5(c) and (d), respectively. From the coercivity and the remanence values in these hysteresis curves, the T_CM_ for Tb2210 is found to be approximately around 250 K where it transforms from Tb dominant sublattice below 250 K to Fe dominant sublattice above it. Increasing the film thickness to 20 nm in Tb2220 results in Tb dominance over the entire range of temperature until it reaches T_c_ at 380 K. We examined the switching direction in samples Tb2210 and Tb2220 and observed the same inversion of switching direction as we observed in samples Tb3010 and Tb3020 at a given helicity (data not shown). Despite the different dominant sublattice at room temperature, presence of compensation temperature and diverse coercivity values, the switching direction seems to only depend on the film thickness. Fig. 5(e) shows all the investigated samples with variable concentrations and thicknesses and their respective switched directions with σ+ helicity measured at Fe *L*_3_ edge. It is indeed remarkable that we observe clear switching in the ring region in films with thickness as high as 80 nm even though the demagnetization energy is expected to increase in thicker films and the attenuation length of the laser is about 20 nm. There have been studies claiming that the switching direction in ferrimagnets depend on the magnetic orientation of an individual sublattice rather than the total magnetization of the film^30^. If we focus at samples with different dominant sublattices and constant film thickness like previous studies, this is confirmed by our observations (Tb3010 and Tb2210). However, the inversion of switching direction above a threshold thickness is rather surprising. It most likely indicates the presence of two competing mechanisms with different dependence on thickness contributing to AO-HDS.

A detailed theoretical explanation of the observed thickness dependence lies beyond the scope of this study, but it might be still worthwhile considering the thickness dependence of the mostly discussed MCD and IFE effects. The MCD effect preferentially demagnetizes one magnetization direction at temperatures close to T_c_ due to a small magnetization dependent difference in the absorption cross section^9, 12–14^. This MCD effect in absorption can only play a role if a significant part of the incoming laser intensity is transmitted. For a film thickness above the attenuation length (about 20 nm for TbFe at a wavelength of 800 nm), when most of the laser power gets absorbed by the TbFe film, the deposited laser energy will start to match the intensity profile of the laser and no longer vary with the orientation of magnetic domains. However, reflectivity also changes with the magnetization direction in presence of MCD. We measured the magnetization dependent changes on transmission and reflectivity for our samples at room temperature, as displayed in Fig. 5(f). We calculated the resulting total MCD effect, in terms of laser energy deposited in the sample, by subtracting the reflected and transmitted parts from the total incoming intensity. We find that the resulting total MCD effect is almost zero for thin films and increases with thickness. This is because the absorption and reflectivity have opposite sign and almost compensate each other for thin films. In thicker films, the magnetization dependent changes in reflectivity dominate, as the contributions of the absorption to the MCD effect vanishes. Unfortunately we were not able to match the conditions in terms of laser fluence and temperature gradients between reflectivity and the previous PEEM measurements. But the observed trend indicates that the contributions of reflectivity to the MCD effect might be responsible for AO-HDS in our thicker TbFe films. The reversed helicity dependence in 10 nm thick films suggest a second mechanism that dominates below the attenuation length of laser light. This could either be the enhanced contributions of absorption to the resulting MCD effect even though we do not observe a sign reversal in the resulting total MCD effect. We do not exclude that this disagreement could be caused by the mentioned differences in the experimental conditions during the reflectivity measurements, but it might as well indicate the presence of a second mechanism such as the IFE that dominates over MCD effects. Unfortunately, the theoretical models on IFE either do not include or do not explain the implications of dissipative systems such as metallic thin films, but the most recent calculations estimate that the momentum transfer by the laser light is too low to switch the magnetization by single pulse^31–33^. Therefore, the IFE is expected to switch the magnetization only near T_c_ where the susceptibility is high enough. However, to understand the direction of the IFE would require detailed calculations on the spin-orbit coupling in the valence band of our system.

To conclude, we have investigated the effect of temperature on AO-HDS in TbFe thin films with different magnetic properties, spatially resolved. We demonstrated that in a Gaussian femtosecond laser spot, the helicity dependent switching is a local phenomenon occurring only at the periphery of thermal magnetization. Our findings are in agreement with previous studies which stress on the importance of attaining T_c_ irrespective of the mechanism proposed for the switching behavior^8, 9, 13, 14^. In addition, we show that helicity dependent switching process depends on thermally activated domain wall motion and the thermal gradient established around the laser spot. We observed a novel effect that the helicity dependence of laser induced switching reverses with thickness independent of any other magnetic property. This indicates the presence of two competing mechanisms contributing to AO-HDS.

## Methods

### Sample preparation

We deposit Tb_x_Fe_1−x_ alloy films with different thickness on a transparent MgO substrate using magnetron sputtering at room temperature. A buffer layer of 5 nm Ta is used to promote a smooth interfacial growth. The samples are capped with 1.8 nm Pt to protect against oxidation. All the samples are magnetized with a permanent magnet before mounting them on the sample holder. As a result, they displayed a homogenously magnetized state by PEEM.

### Laser setup

For laser excitation we used a Femtolasers Scientific XL Ti:Sapphire oscillator with a central wavelength of 800 nm. It provides variable pulse duration from 80–500 fs with an adjustable repetition rate ranging from 2.5 MHz to a single laser pulse. A dedicated sample holder with integrated laser optics and temperature control is used. After being reflected from a mirror and focused through a lens, the laser is focused at normal incidence onto the backside of the TbFe film^34^. A λ/4 plate is used in the experimental set up to obtain circularly polarized laser pulses, *σ*+ and *σ*−. The total fluence reached to the sample is assumed to undergo a loss of approximately 50% due to the reflections and absorptions from the mirrors and lenses used in our measurement set up. We used a Ti:Sapphire oscillator with a central wavelength of 800 nm operating at 80 MHz for reflectivity and transmission measurements. Reflectivity and transmission was recorded as a function of magnetic field and laser helicity for different sample thicknesses.

### SQUID measurements

The hysteresis loops are measured at different temperature using a Quantum Design SQUID-VSM in the out of plane configuration. The remanent magnetization values at different temperatures are extracted from the corresponding hysteresis measurements.

## Electronic supplementary material

Supplementary Information
