# Supplementary material for: Spatially resolved investigation of all optical magnetization switching in TbFe alloys
Source: Sci Rep. 2017 Aug 25;7:9456. doi: 10.1038/s41598-017-09615-1 (PMC5573396; doi:10.1038/s41598-017-09615-1)
Supplement: Supplementary file 1 — Supplementary Information [file 41598_2017_9615_MOESM1_ESM.pdf]

Spatially resolved investigation of all optical magnetization switching in TbFe alloys

Ashima Arora[1], Mohamad-Assaad Mawass[1], Oliver Sandig[2], Chen Luo[3], Ahmet A. Ünal[1, 4], Florin Radu[1], Sergio Valencia[1], Florian Kronast[1]*

[1] *Helmholtz-Zentrum Berlin für Materialien und Energie, Albert-Einstein Str. 15, 12489 Berlin, Germany*

[2] *Institut für Experimentalphysik, Freie Universität Berlin, Arnimallee 14, 14195 Berlin, Germany*

[3] *University of Regensburg, Universitätsstraße 31, 93053 Regensburg, Germany*

[4] *Present address: Max Born Institute for Nonlinear Optics and Short Pulse Spectroscopy, Berlin, Germany*

**Corresponding author: E-mail address: ashima.arora@helmholtz-berlin.de*

Sample name	Dominant sublattice	Compensation Temperature
**Tb2210**	**Fe**	**250 K**
**Tb2220**	**Tb**	×
**Tb2240**	**Tb**	×
**Tb2280**	**Tb**	×
**Tb3010**	**Tb**	×
**Tb3020**	**Tb**	×

Figure S1: Table showing the names of the measured samples, their dominant sublattice and compensation temperature

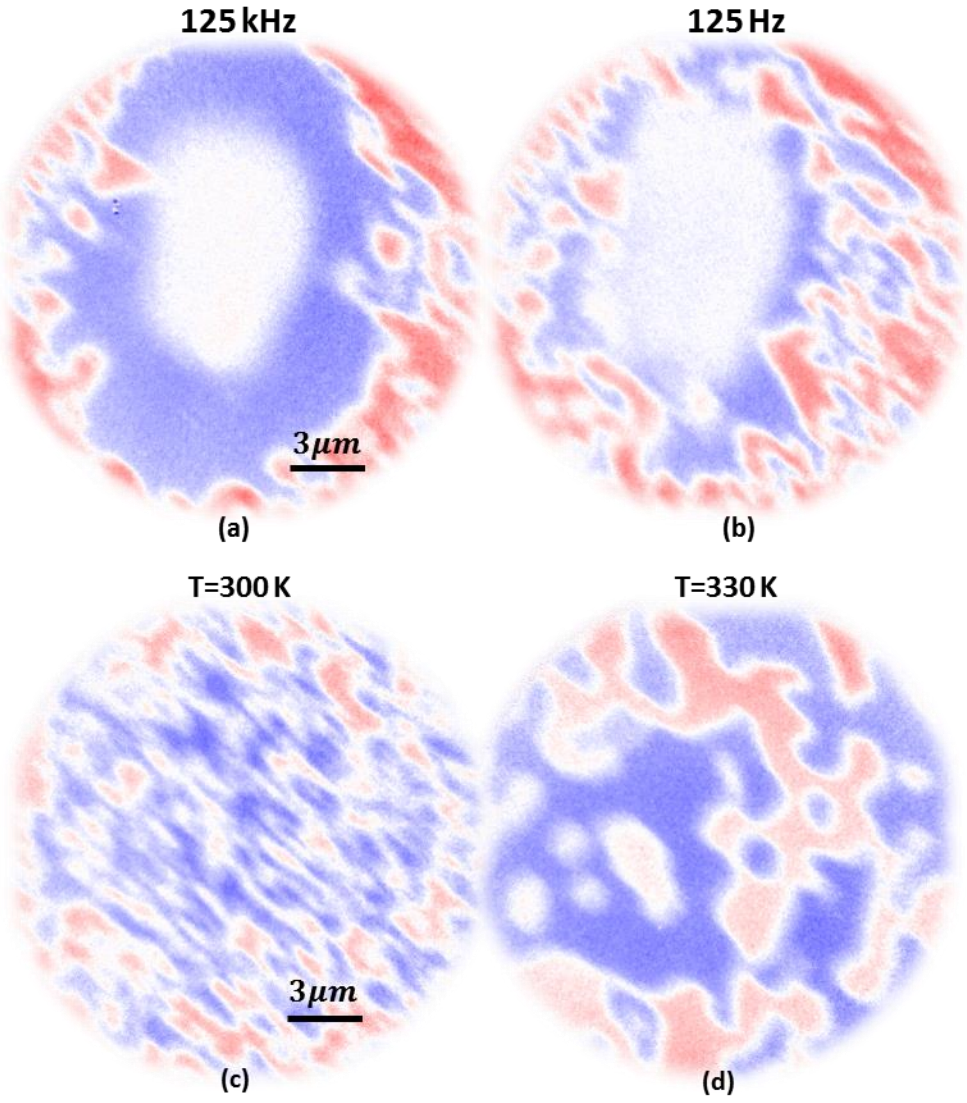

Figure S2: At constant base temperature and laser fluence, repetition rate of the laser plays a vital role in regulating the local temperature profile. Figure S2 (a) and S2 (b) show the XMCD images displaying AO-HDS in the ring region with same helicity and laser fluence but with a different repetition rate of 125 kHz and 125 Hz, respectively. Although the temperature increase per pulse in both the cases is same, a higher thermal gradient across the laser spot is responsible for thermally activated domain wall motion to farther lateral distances. Figure S2 (c) show magnetic domains after a single laser pulse at 300 K. Figure 2 (d) show the same area as shown in S2 (c) at 330 K. The sample was not exposed to the laser at 330 K.
